# Perioperative management of a patient with retrosternal giant goiter and Gitelman syndrome: a case report

**DOI:** 10.3389/fmed.2026.1920327

**Published:** 2026-08-26

**Authors:** Dongdong Chen, Chen Li, Chengming Zhang, Wenwen Wang

**Affiliations:** 1 Department of Anesthesiology, The Affiliated Hospital of Shandong Second Medical University, Weifang, China; 2 Department of Anesthesiology, The 960th Hospital of PLA, Jinan, China; 3 School of Anesthesiology, Shandong Second Medical University, Weifang, China

**Keywords:** anesthesia management, Gitelman syndrome, hypokalemia, perioperative management, retrosternal goiter

## Abstract

**Background:**

Gitelman syndrome is an autosomal recessive renal tubular disorder caused by mutations in the SLC12A3 gene, with the pathogenic mechanism being impaired function of the thiazide-sensitive sodium-chloride cotransporter. The disease is mainly characterized clinically by hypokalemia, hypomagnesemia, metabolic alkalosis, and hypocalciuria. Retrosternal giant goiter is mostly caused by nodular goiter enlarging and extending downward into the anterior mediastinum, which may lead to tracheal compression and displacement. Patients with retrosternal giant goiter complicated with Gitelman syndrome often present with severe electrolyte disturbances and risk of difficult airway, and relevant literature reports are relatively rare.

**Case summary:**

This article reports a 75-year-old male patient with Gitelman syndrome who was scheduled for thyroidectomy due to “retrosternal giant goiter causing left recurrent laryngeal nerve palsy.” Laboratory examination revealed severe hypokalemia, hypomagnesemia, hyponatremia, hypochloremia, and metabolic alkalosis. The supine hypertension panel showed significantly elevated renin activity. Ambulatory electrocardiography suggested frequent premature atrial contractions (>14,000 beats/24 h). Imaging revealed a huge mass in the left thyroid lobe (6.6 cm × 4.1 cm) extending into the mediastinum, compressing the trachea and esophagus, with right-sided aortic arch deformity. After multidisciplinary consultation and active correction of electrolyte disturbances, the patient successfully underwent left total thyroidectomy + recurrent laryngeal nerve exploration under general anesthesia. Key management points included: preoperative optimization of electrolytes to safe thresholds; avoidance of drugs that prolong the QT interval and affect electrolyte balance; intraoperative continuous electrocardiographic monitoring and electrolyte monitoring; and formulation of a difficult airway plan. The patient recovered well postoperatively without serious complications such as arrhythmias.

**Conclusion:**

The perioperative management of retrosternal giant goiter complicated with Gitelman syndrome is highly challenging. In this case, adequate preoperative assessment, active correction of electrolyte disturbances to safe thresholds, rational selection of anesthetic agents, formulation of a difficult airway management plan, and close monitoring were associated with a favorable clinical outcome.

## 1 Introduction

Gitelman syndrome (GS) is a rare autosomal recessive renal tubular disorder caused by mutations in the SLC12A3 gene, which leads to impaired function of the thiazide-sensitive sodium-chloride cotransporter (NCC) and consequently results in defective sodium and chloride reabsorption in the distal renal tubule, with a prevalence of approximately 1 in 40,000. Its laboratory findings are classically characterized by hypokalemia, hypomagnesemia, metabolic alkalosis, and hypocalciuria, often accompanied by activation of the renin-angiotensin-aldosterone system (RAAS) (1, 2). Clinical manifestations include muscle weakness, fatigue, muscle cramps, thirst, and nocturia, and in severe cases, life-threatening conditions such as arrhythmias and sudden death may occur (3, 4). Retrosternal goiter is defined as a goiter in which more than 50% of the thyroid mass is located below the thoracic inlet; it may compress the trachea, esophagus, and major cervical vessels, leading to symptoms such as dyspnea and dysphagia. The risks of difficult airway, intraoperative bleeding, and postoperative airway obstruction during the perioperative period are relatively high. To date, literature reports on the perioperative management of patients with retrosternal giant goiter complicated with Gitelman syndrome are rare. This article reports a male patient with retrosternal giant goiter accompanied by left recurrent laryngeal nerve palsy and concurrent Gitelman syndrome, who successfully underwent anesthesia and surgery through multidisciplinary collaboration. We also review the latest literature to explore the perioperative management strategies for this case, with the aim of emphasizing the importance for anesthesiologists and nursing staff of recognizing and managing Gitelman syndrome.

## 2 Case report

### 2.1 General information

The patient was a 75-year-old male, 170 cm in height and 68 kg in weight, who was admitted to the hospital due to “discovery of a left thyroid mass with hoarseness for 20 days.” The patient reported no pain, dyspnea, dysphagia, or palpitations. His past medical history was unremarkable, with no history of hypertension, diabetes mellitus, or cardiac disease, and no history of drug or food allergies. The patient was independent in activities of daily living, with a modest financial status, no history of psychiatric disorders, harmonious family relationships, and good social support. A definite diagnosis of Gitelman syndrome had been documented prior to admission, and the clinical features were also highly consistent with the diagnosis; however, the patient had not received regular treatment, and genetic testing had not been performed. After admission, multidisciplinary consultations were obtained, and electrolyte disturbances were actively corrected according to the consultation recommendations.

### 2.2 Physical examination

Body temperature was 36.5 °C pulse rate 78 beats/min, respiratory rate 18 breaths/min, and blood pressure 136/80 mmHg. The patient was alert and oriented. The neck was soft, with the trachea deviated to the right. A mass of approximately 6 cm × 6 cm was palpable in the left anterior cervical region; it was firm, well-defined, non-tender, and moved with swallowing. No obvious mass was palpated in the right thyroid lobe. No significant enlarged lymph nodes were palpable in the neck. Breath sounds were clear in both lungs, with no dry or moist rales. The heart rate was 78 beats/min with an irregular rhythm, and no pathological murmurs were heard in any valve auscultation areas.

### 2.3 Auxiliary examinations

#### 2.3.1 Laboratory tests

Admission complete blood count showed: white blood cell (WBC) count 6.26 × 10^9^/L (↑), red blood cell (RBC) count 3.56 × 10^12^/L (↓), hemoglobin (HGB) 117 g/L (↓), hematocrit (HCT) 0.313 (↓), mean corpuscular hemoglobin concentration (MCHC) 374 g/L (↑), monocyte percentage (MONO%) 11.0% (↑), eosinophil percentage (EO%) 3.9% (↑), basophil percentage (BASO%) 0.5% (↑), with other parameters within normal limits. Lipid profile showed: total cholesterol (CHOL) 5.73 mmol/L (↑), triglycerides (TG) 1.85 mmol/L (↑). Perioperative biochemical tests consistently indicated severe electrolyte disturbances (Table 1). Thyroid function tests showed thyroglobulin >500 ng/mL (↑), with other parameters within normal limits. Liver function tests showed alanine aminotransferase 63.5 U/L, aspartate aminotransferase 41.8 U/L, and gamma-glutamyl transferase 108.2 U/L; renal function was normal. Supine hypertension panel and 24-h urinary electrolyte tests showed partial abnormalities and all abnormalities, respectively (Table 2).

**Table 1:** Perioperative electrolyte measurements.

Test item	Day 1	Day 4	Day 8	Day 12	Day 13 (Intraoperative ABG)	Day 14	Reference range
K^+^	2.08	2.52	2.69	3.29	3.1	3.38	3.5 ~ 5.3 mmol/L
Mg^2+^	/	0.51	0.44	0.48	/	0.58	0.75 ~ 1.02 mmol/L
Na^+^	133	135	140	141	133	135	137 ~ 147 mmol/L
Cl^−^	83.5	86.6	94.4	100.3	100	101	99 ~ 110 mmol/L
Ca^2+^(Total Ca)	2.03	/	2.08	1.97	0.98(iCa)	2.01	2.11 ~ 2.52 mmol/L
Phos	/	1.21	1.19	1.14	/	1.16	0.85 ~ 1.51 mmol/L
HCO_3_^~^	41	42.8	36.7	31.6	32.4	31.3	20 ~ 29 mmol/L

**Table 2:** Supine hypertension panel and 24-h urinary electrolytes.

Test item	Hypertension panel (supine)			24-h urinary electrolytes				
	Renin activity (ng/mL/h)	Aldosterone (ng/dL)	Angiotensin II(pg/mL)	Urinary K^+^ (mmol/24 h)	Urinary Na^+^ (mmol/24 h)	Urinary Cl^−^ (mmol/24 h)	Urinary Ca^2+^ (mmol/24 h)	Urinary P (mmol/24 h)
Result	12.49	4.22	107.50	128.33	312.40	298.32	2.40	12.45
Reference range	0.15 ~ 2.33	3 ~ 16	25 ~ 60	25 ~ 100	130 ~ 260	170 ~ 250	2.5 ~ 7.5	22–48

#### 2.3.2 Electrocardiography and echocardiography

Electrocardiography showed sinus rhythm, premature atrial contractions, and incomplete right bundle branch block. Ambulatory electrocardiography revealed sinus rhythm, frequent premature atrial contractions (14,272 beats/24 h), paired and short runs of atrial tachycardia, occasional premature ventricular contractions, and incomplete right bundle branch block. Color Doppler echocardiography demonstrated mild aortic regurgitation, mild tricuspid regurgitation, mild mitral regurgitation, and mild pulmonary regurgitation, with an estimated pulmonary artery systolic pressure of 40 mmHg (mild pulmonary hypertension).

#### 2.3.3 Imaging examinations

**Figure 1:**
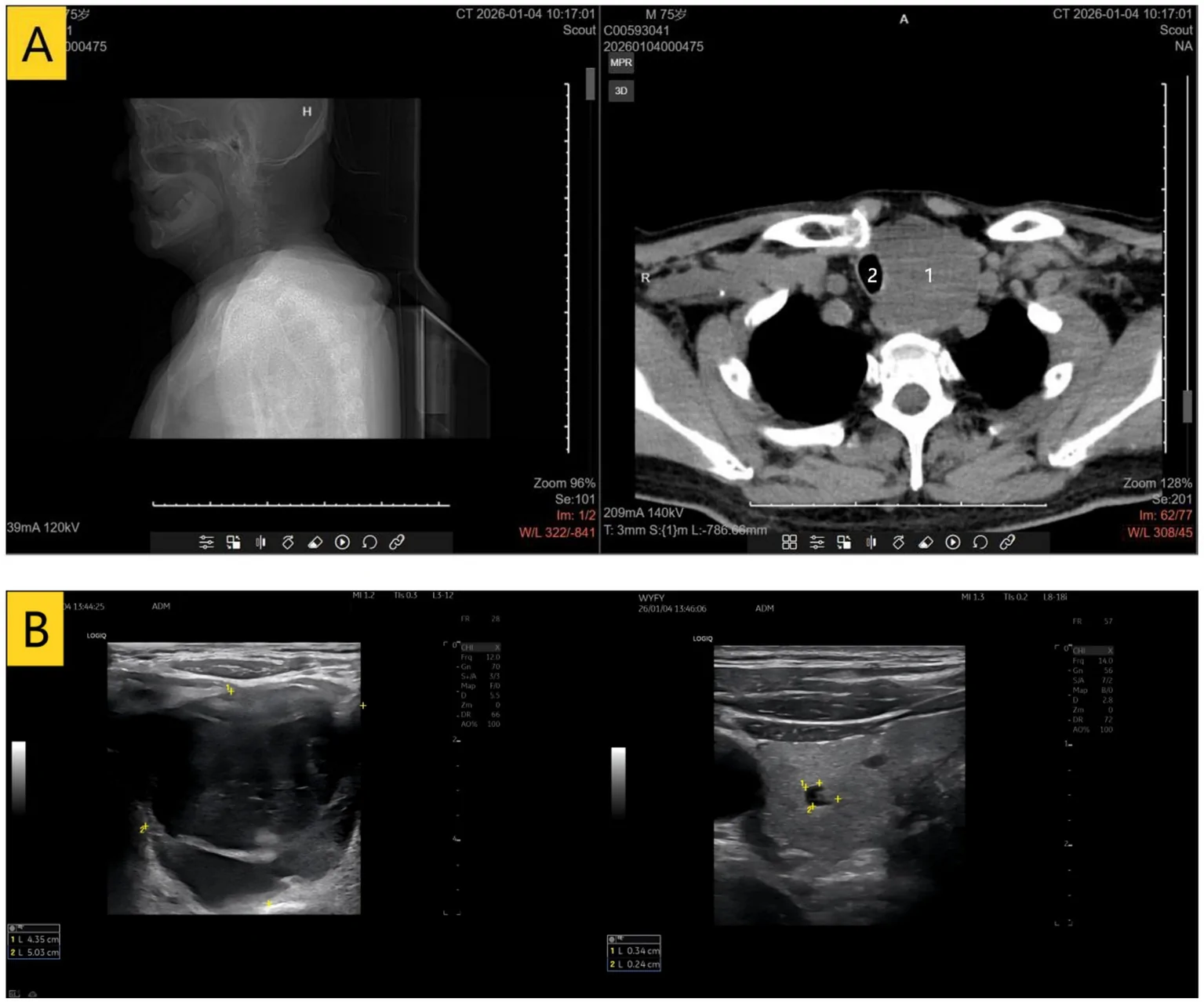
Imaging findings of the patient with Gitelman syndrome. **(A)** Enlargement of the left thyroid lobe with downward extension into the mediastinum, showing a roundish slightly hypodense area; the adjacent trachea and esophagus are compressed and displaced, with unclear demarcation in some areas. **(B)** Cystic-solid nodules in both thyroid lobes.

Contrast-enhanced CT of the neck showed enlargement of the left thyroid lobe extending downward into the mediastinum, with a roundish slightly hypodense area measuring approximately 6.6 cm × 4.1 cm, causing compression and displacement of the adjacent trachea and esophagus, with unclear demarcation in some areas (Figure 1A). The aortic arch was located on the right side (right-sided aortic arch deformity). Color Doppler ultrasound of the thyroid and cervical lymph nodes revealed cystic-solid nodules in both thyroid lobes (C-TIRADS grade 3), with the largest in the left lobe measuring approximately 4.3 cm × 5.0 cm and predominantly cystic (Figure 1B).

### 2.4 Perioperative management

#### 2.4.1 Preoperative preparation

After admission, multidisciplinary consultations were obtained from the Departments of Nephrology, Cardiology, Endocrinology, Metabolic Disease, Rehabilitation Medicine, and Clinical Nutrition. Recommendations included: (1) active correction of electrolyte disturbances with intravenous and oral potassium and magnesium supplementation; (2) completion of ambulatory electrocardiography for assessment and administration of metoprolol succinate to control arrhythmias; (3) increased dietary intake of potassium and magnesium-rich foods; (4) administration of potassium-sparing diuretics if necessary; and (5) elective surgery after electrolyte levels were substantially corrected. Based on the comprehensive consultation opinions, the patient received daily intravenous infusion of 500 mL glucose-sodium chloride solution containing 1 g concentrated sodium chloride and 5 mL of 15% potassium chloride, along with oral calcium carbonate D3 tablets 0.6 g/day and potassium magnesium aspartate 3 tablets/day before and on the day of surgery to actively correct electrolyte disturbances. After 10 days of correction, the electrolyte disturbances improved significantly (Table 1). Following preoperative ambulatory electrocardiography assessment, metoprolol succinate was added to improve arrhythmias. Potassium and magnesium supplementation was continued before and on the day of surgery. Routine preoperative fasting and fluid restriction were observed, and phenobarbital sodium 0.1 g was administered intramuscularly half an hour before surgery.

#### 2.4.2 Anesthetic induction and perioperative management

The patient had a Mallampati class II, mouth opening of 4 cm, thyromental distance of 6 cm, and neck extension >80°, with tracheal deviation to the right. Imaging of the neck revealed compression and displacement of the trachea and esophagus; therefore, a potentially difficult airway could not be excluded in this patient, and a detailed difficult airway management plan was therefore formulated by the Department of Anesthesiology. Anesthesia induction employed a strategy of awake intubation with sedation, with a fiberoptic bronchoscope and various sizes of endotracheal tubes prepared. In selecting anesthetic agents, drugs that might prolong the QT interval or induce hypokalemia were avoided.

Before anesthetic induction, ABP was 120 ~ 135/60 ~ 65 mmHg with HR 70 ~ 75 bpm. Anesthesia induction was initiated with intravenous midazolam 2 mg and a loading dose of dexmedetomidine 50 μg via infusion pump, combined with adequate topical anesthesia of the oropharynx, tongue base, and laryngopharynx using 1% lidocaine. Subsequently, an endotracheal tube (size 6.5) was placed under video fiberoptic bronchoscopic guidance to the appropriate position and securely fixed. After intubation, ABP was 150/70 mmHg with HR 70 bpm. Then, sufentanil 20 μg, etomidate 15 mg, and rocuronium 50 mg were administered intravenously, and the patient was switched to mechanical ventilation with appropriate parameters set. Five minutes after induction, ABP dropped to 70/40 mmHg with HR 62 bpm. Dopamine was started at 6 ~ 8 μg/kg/min via continuous infusion, after which ABP was maintained at 105 ~ 145/55 ~ 60 mmHg with HR 65 ~ 80 bpm. Intraoperative monitoring included continuous electrocardiography, pulse oximetry, invasive arterial blood pressure, end-tidal carbon dioxide partial pressure, and bispectral index (BIS) monitoring. Anesthesia was maintained with continuous intravenous infusion of propofol at 3 ~ 5 mg/kg/h and remifentanil at 0.3 ~ 0.5 μg/kg/min, with intermittent boluses of rocuronium 15 mg administered 1 h after the start of surgery. Intraoperative correction of electrolyte disturbances was primarily achieved with 0.9% normal saline; peripheral intravenous potassium supplementation was administered at a concentration ≤3‰ and a rate ≤0.15 mmol/kg/h, with a target serum potassium of 3.5 ~ 4.0 mmol/L, combined with intravenous magnesium sulfate infusion; in cases of hypocalcemia, 10% calcium gluconate was diluted and slowly injected. Arterial blood gases and electrolytes were monitored throughout. The surgical procedure was left total thyroidectomy with recurrent laryngeal nerve exploration, lasting approximately 2 h. Intraoperative blood loss was approximately 10 mL, and fluid infusion was 1,000 mL.

#### 2.4.3 Postoperative management

At the end of surgery, sugammadex 140 mg was administered. After the patient regained full consciousness, muscle strength recovered, and spontaneous breathing and tidal volume reached target levels, a trial extubation procedure was performed: the endotracheal tube was withdrawn to a position above the tracheal stenosis segment, and the patient was observed for 10 min. No airway obstruction manifestations such as chest tightness, dyspnea, or triple retraction sign were observed. Combined with intraoperative findings excluding tracheomalacia, and confirmation of airway patency, the endotracheal tube was successfully removed. After extubation, the patient’s vital signs were stable; hoarseness was unchanged from preoperative status; there was no dyspnea, perioral numbness, or tetany; and no significant oozing from the incision was observed. The patient was subsequently transferred to the general ward. Continuous electrocardiographic monitoring, drainage tube management, nebulization therapy, and symptomatic supportive care were continued. Potassium and magnesium supplementation was maintained. The patient recovered well postoperatively, with hoarseness showing no significant improvement compared with before surgery; no dyspnea, tetany, or wound infection occurred. Postoperative pathology revealed: (left thyroid) nodular goiter with hemorrhage and cystic change, with focal lymphocytic infiltration and multinucleated giant cell reaction, and a few epithelioid-like cell atypical hyperplasia. The patient was discharged on postoperative day 5, with instructions to regularly follow up electrolyte levels and thyroid function.

#### 2.4.4 Postoperative follow-up

The patient was discharged on postoperative day 5, with discharge instructions including wound protection, thyroid function re-examination at 6 weeks, and monitoring of electrolyte changes. At 2 months after discharge, a telephone follow-up was conducted. The patient reported continuing oral potassium chloride and potassium magnesium aspartate, with no significant fatigue, muscle cramps, or palpitations, and no limitation in daily activities. The patient expressed satisfaction with the surgical outcome.

Patient statement: “I found a large mass in my thyroid because of hoarseness, and the doctors said I needed surgery. However, laboratory tests revealed severe deficiencies in potassium, magnesium, and other electrolytes in my body, along with premature atrial contractions, so the surgery was postponed. Later, doctors from multiple departments held a consultation and prescribed intravenous and oral electrolyte supplementation. After 10 days of supplementation, my electrolyte levels approached the safe range, and the surgical team decided to proceed with the operation. The anesthesiologist told me that awake intubation was planned and asked me to cooperate; however, I have no memory of the entire procedure and experienced no significant discomfort. The surgery went smoothly, and I was discharged on the fifth day after surgery. Since discharge, I have been taking electrolyte supplements as instructed by the doctors and have had no significant fatigue or cramps. Although my hoarseness remains unchanged, I can accept it. I am very satisfied with the treatment process and outcome, and I am grateful to all the doctors and nurses involved in my care.”

### 2.5 Perioperative timeline

The patient’s clinical course from admission to discharge is presented in Table 3. It shows the sequential changes in serum electrolytes, the timing of multidisciplinary consultations, the day of surgery, and the postoperative recovery period.

**Table 3:** Perioperative timeline and key laboratory findings.

Time point	Event	Key findings
Day 1 (admission)	Admitted for left vocal cord paralysis and goiter; multidisciplinary consultation initiated	K^+^ 2.08 mmol/L, HCO_3_^−^ 41 mmol/L; CT: tracheal compression, right-sided aortic arch; ECG: frequent PACs (>14,000/24 h)
Day 4	Continued electrolyte correction; consulted nephrology and cardiology	K^+^ 2.52 mmol/L, Mg^2+^ 0.51 mmol/L, HCO₃^−^ 42.8 mmol/L; 24 h urine K^+^ 128.33 mmol
Day 8	Electrolytes still low; consulted endocrinology and metabolic disease	K^+^ 2.69 mmol/L, Mg^2+^ 0.44 mmol/L, HCO₃^−^ 36.7 mmol/L
Day 12	Electrolytes approaching target; surgery scheduled for next day	K^+^ 3.29 mmol/L, Mg^2+^ 0.48 mmol/L, HCO_3_^−^ 31.6 mmol/L
Day 13 (surgery)	Left total thyroidectomy + recurrent laryngeal nerve exploration	Intraoperative: K^+^ 3.1 mmol/L, ionized Ca^2+^ 0.98 mmol/L; hypotension 70/40 mmHg after induction
Day 14	Postoperative day 1	K^+^ 3.38 mmol/L, Mg^2+^ 0.58 mmol/L, Ca^2+^ 2.01 mmol/L, HCO₃^−^ 31.3 mmol/L
Day 15–16	Recovery	No dyspnea, hoarseness unchanged
Day 17(charge)	Discharged	Electrolytes not rechecked, Follow-up advised

## 3 Discussion

This patient was a 75-year-old male with retrosternal giant goiter complicated by left recurrent laryngeal nerve palsy and right-sided aortic arch deformity, presenting with severe hypokalemia (nadir 2.08 mmol/L), hypomagnesemia (nadir 0.44 mmol/L), hyponatremia, hypochloremia, metabolic alkalosis (peak HCO_3_^−^ 42.8 mmol/L), elevated renin activity (12.49 ng/mL/h), and hypocalciuria (2.40 mmol/24 h). The perioperative period was confronted with major risks including GS-related severe electrolyte disturbances, airway compression by the retrosternal giant goiter, and arrhythmias. Herein, we analyze the perioperative management difficulties and strategies for this case in the context of the literature (Table 3).

### 3.1 Diagnosis and differential diagnosis

According to the Gitelman syndrome consensus guidelines (1), this patient met the following diagnostic criteria: (1) chronic hypokalemia (serum potassium < 3.5 mmol/L) with renal potassium wasting (urinary potassium 128.33 mmol/24 h); (2) metabolic alkalosis (peak HCO_3_^−^42.8 mmol/L); (3) hypomagnesemia (< 0.7 mmol/L); (4) hypocalciuria (urinary calcium < 2.5 mmol/24 h); (5) elevated renin activity. The clinical and biochemical features were highly consistent with the diagnosis of Gitelman syndrome.

In terms of differential diagnosis, the following conditions should be considered: (1) Bartter syndrome, which typically presents in infancy with hypercalciuria and nephrocalcinosis—inconsistent with the adult onset and hypocalciuria in this patient; (2) diuretic or laxative abuse, which was excluded by the absence of relevant medication history and the urinary electrolyte profile; (3) primary aldosteronism, which is characterized by suppressed renin activity rather than elevated levels—a pattern not observed in this patient; and (4) Liddle syndrome, which presents with hypertension, hypokalemia, and low renin levels—contrasting with the hypotension and elevated renin activity seen in this case. In summary, the clinical diagnosis of Gitelman syndrome was well established.

### 3.2 Goals and strategies for perioperative electrolyte management

Chronic hypokalemia and hypomagnesemia may lead to myocardial repolarization abnormalities, and approximately 50% of GS patients have a prolonged QTc interval, increasing the risk of torsade de pointes (TdP) and sudden cardiac death (5, 6). In this patient, ambulatory electrocardiography revealed frequent premature atrial contractions and short runs of atrial tachycardia. Although no ventricular arrhythmias were documented, he remained at high risk for anesthesia.

The KDIGO consensus guidelines recommend that preoperative serum potassium should be maintained at ≥3.0 mmol/L and serum magnesium at ≥0.6 mmol/L in GS patients (1). On admission, this patient had a serum potassium of 2.08 mmol/L and serum magnesium of 0.51 mmol/L. After intravenous and oral potassium and magnesium supplementation, the preoperative serum potassium rose to 3.29 mmol/L and serum magnesium to 0.48 mmol/L (still below target). Based on the patient’s retrosternal giant goiter causing tracheal compression and displacement, delayed surgery carried a risk of progressive airway compromise. Given that the serum magnesium level remained relatively stable without associated clinical symptoms of hypomagnesemia, and the serum potassium had reached the safe threshold, intraoperative real-time electrolyte monitoring and intravenous magnesium supplementation were feasible. After comprehensive multidisciplinary assessment, the decision to proceed with surgery was made. Intraoperative arterial blood gas analysis was continuously monitored, and the intravenous potassium infusion rate was adjusted accordingly. Postoperative supplementation was continued. In this patient, no severe intraoperative or postoperative arrhythmias were observed. Magnesium deficiency impairs renal potassium reabsorption; therefore, aggressive magnesium supplementation should be undertaken in GS patients to improve renal potassium retention (7). This strategy may be considered in comparable cases, with its suitability to be determined according to individual clinical circumstances.

### 3.3 Selection of anesthetic agents and circulatory management

Patients with GS, due to chronic renal potassium and magnesium wasting, are in a state of systemic potassium and magnesium depletion, accompanied by pathophysiological alterations including abnormal angiotensin II signaling, upregulation of the nitric oxide system, and microvascular dysfunction (3). Myocardial cells are extremely sensitive to fluctuations in extracellular potassium concentrations. The depolarizing muscle relaxant succinylcholine can induce muscle fiber depolarization, leading to intracellular potassium efflux and a relative increase in serum potassium, which may precipitate TdP or even cardiac arrest. Antiemetics such as droperidol can significantly prolong the QTc interval and should be strictly avoided (1, 8). In addition, hypokalemia potentiates the neuromuscular blocking effects of both depolarizing muscle relaxants such as succinylcholine and non-depolarizing muscle relaxants such as rocuronium and vecuronium, increasing the need for postoperative ventilatory support (9, 10). In this case, rocuronium was selected as the neuromuscular blocking agent, and its effects were reversed at the end of surgery with the specific antagonist sugammadex. No complications related to residual neuromuscular blockade were observed. Invasive arterial blood pressure was continuously monitored throughout the procedure. The patient developed severe hypotension 5 min after induction, which was rapidly reversed with dopamine at 6–8 μg/kg/min. This dose is within the standard therapeutic range for intraoperative hypotension. After administration, blood pressure rose steadily without significant tachycardia, suggesting good tolerance in this patient. However, whether this regimen is applicable to other patients with Gitelman syndrome still requires comprehensive evaluation based on individual volume status, cardiac function, and intraoperative monitoring conditions, and should not be directly generalized.

### 3.4 Airway and volume management

Retrosternal giant goiter can compress the trachea, causing narrowing, deviation, or even softening of the airway, significantly increasing the difficulty of airway management. Preoperative CT in this patient showed compression and displacement of the trachea and esophagus, strongly suggesting the possibility of a difficult airway. It has been reported in the literature that the incidence of tracheal deviation in patients with retrosternal goiter can reach 62.5%, and tracheal compression 43.8%, with the degree of compression being significantly correlated with postoperative dyspnea and prolonged ventilatory support (11). Awake fiberoptic bronchoscopic intubation is the gold standard for difficult airway management, but most patients with retrosternal goiter can be successfully intubated with direct laryngoscopy or video laryngoscopy after conventional intravenous induction. In addition, postoperative vigilance is required for tracheomalacia, which occurs in approximately 15.6% of patients with retrosternal goiter (12), primarily manifesting as airway collapse and dyspnea after extubation. In this case, no significant tracheomalacia was observed postoperatively, which may be related to the intraoperative exploration that excluded tracheomalacia and the trial extubation strategy. However, the outcome of a single case should not be used as a routine recommendation. Whether to adopt a similar strategy should be determined individually based on intraoperative findings and the patient’s airway condition. Regarding volume management, patients with Gitelman syndrome often have relative hypovolemia due to chronic sodium loss and RAAS activation, rendering them prone to hypotension during anesthesia induction (8). In this case, an appropriate crystalloid preload was administered before induction, and induction agents were given slowly in divided doses to maintain hemodynamic stability.

### 3.5 Limitations

The clinical presentation and biochemical features of this patient were highly consistent with the diagnostic criteria for Gitelman syndrome; however, SLC12A3 genetic testing was not performed, and thus molecular genetic confirmation is lacking. The postoperative follow-up period was relatively short, and the patient’s electrolyte control status and long-term prognosis after discharge remain unclear, necessitating further long-term follow-up. As a single case report, the conclusions are subject to inherent selection bias and publication bias, and cannot be generalized to all GS patients with retrosternal goiter. Future multicenter, prospective studies with genetically confirmed diagnoses and larger sample sizes are needed to further define optimal perioperative management strategies for this patient population.

## 4 Conclusion

The perioperative management of patients with retrosternal giant goiter and Gitelman syndrome is highly challenging and requires multidisciplinary collaboration and an individualized anesthesia plan. In the present case, a favorable clinical outcome was associated with adequate preoperative assessment, aggressive correction of electrolyte disturbances to safe thresholds, rational selection of anesthetic agents, formulation of a difficult airway management plan, and close perioperative monitoring. Nevertheless, further studies with larger sample sizes are required to confirm the general applicability of these management strategies.

## Data Availability

The original contributions presented in the study are included in the article/supplementary material, further inquiries can be directed to the corresponding authors.
